# Supplementary material for: Low Inflammatory Stimulus Increases D2 Activity and Modulates Thyroid Hormone Metabolism during Myogenesis In Vitro
Source: Metabolites. 2022 May 6;12(5):416. doi: 10.3390/metabo12050416 (PMC9144220; doi:10.3390/metabo12050416)
Supplement: Supplementary file 1 [file metabolites-12-00416-s001.zip › metabolites-1655215-supplementary.pdf]

metabolites

Table S1. Primers sequences.

Gene	**Forward sequence**	**Reverse sequence**	**GenBank accession n°**	**References**
Hprt1	GCAGTACAGCCCCAAAATGG	AACAAAGTCGGCCTGTATCCAA	NM_013556.2	[19]
G6pdx	CATGAGTCAGACAGGCTGGA	GATCTGGTCCTCACGGAAAA	NM_008062.2	[19]
Dio2	GCTTCCTCCTAGATGCCTACAA	CCGAGGCATAATTGTTACCTG	NM_010050.3	[19]
Myod1	GACCTGCGCTTTTTTGAGGACC	CAGGCCCACAGCAAGCAGCGAC	NM_010866.2	[13]
Myog	TTGCTCAGCTCCCTCAACCAGGA	TGCAGATTGTGGGCGTCTGTAGG	NM_031189.2	[19]
Myh4	CACCTGGACGATGCTCTCAGA	GCTCTTGCTCGGCCACTCT	NM_010855.3	[19]
Myh7	ACTGTCAACACTAAGAGGGTCA	TTGGATGATTTGATCTTCCAGGG	NM_080728.3	[19]
Hr	GCAGGTAGTAGAACGGAAGATCC	CTGGTGACAATGGAAGGCTCA	NM_021877.3	[19]
Slc16a2	CGGCTGGATAGTGGTGTTTG	TGGAGTAGAGGATACCAACAGAG	NM_009197.2	PrimerBank
Thra1	GGTCACCAGATGGAAAGCGAA	CCTTGTCCCCACACACGAC	NM_178060	PrimerBank
